# Complete Freund's adjuvant induces experimental autoimmune myocarditis by enhancing IL‐6 production during initiation of the immune response

**DOI:** 10.1002/iid3.155

**Published:** 2017-03-13

**Authors:** Jillian A. Fontes, Jobert G. Barin, Monica V. Talor, Natalie Stickel, Julie Schaub, Noel R. Rose, Daniela Čiháková

**Affiliations:** ^1^ W. Harry Feinstone Department of Molecular Microbiology and ImmunologyJohns Hopkins University Bloomberg School of Public HealthBaltimoreMDUSA; ^2^ Division of Immunology, Department of Pathology, Johns Hopkins UniversitySchool of MedicineBaltimoreMDUSA; ^3^ Department of Hematology, Oncology and Stem Cell TransplantationFreiburg University Medical CenterFreiburgGermany; ^4^ Faculty of BiologyAlbert Ludwigs University FreiburgFreiburgGermany

**Keywords:** Autoimmunity, myocarditis, adjuvant

## Abstract

**Introduction:**

Complete Freund's Adjuvant (CFA) emulsified with an antigen is a widely used method to induce autoimmune disease in animal models, yet the contribution of CFA to the immune response is not well understood. We compared the effectiveness of CFA with Incomplete Freund's Adjuvant (IFA) or TiterMax Gold Adjuvant (TMax) in experimental autoimmune myocarditis (EAM) in male mice.

**Methods:**

EAM was induced in A/J, BALB/c, and IL6KO BALB/c male mice by injection of the myocarditogenic peptide in CFA, IFA, or TMax on days 0 and 7. EAM severity was analyzed by histology on day 21. In addition, specific flow cytometry outcomes were evaluated on day 21.

**Results:**

Only mice immunized with CFA and myocarditogenic peptide on both days 0 and 7 developed substantial myocarditis as measured by histology. We observed a significantly increased level of IL6 in the spleen 3 days after CFA immunization. In the spleen and heart on day 21, there was an expansion of myeloid cells in CFA‐immunized mice, as compared to IFA or TMax‐immunized animals. Recombinant IL‐6 at the time of IFA immunization partially restored susceptibility of the mice to EAM. We also treated EAM‐resistant IL‐6 knockout mice with recombinant IL‐6 around the time of the first immunization, on days −1 to 2, completely restoring disease susceptibility, showing that the requirement for IL‐6 coincides with primary immunization. Examining APC populations in the lymph node draining the immunization site evidenced the contribution of IL‐6 to the CFA‐dependence of EAM was through controlling local dendritic cell (DC) trafficking.

**Conclusions:**

CFA used with myocarditogenic peptide twice is required to induce EAM in both A/J and Balb/c mice. Although IFA and TiterMax induce antibody responses, only CFA preferentially induced autoantigen‐specific responses. CFA expands monocytes in the heart and in the spleen. IL‐6 signaling is required during short window around primary immunization to induce EAM. In addition, IL‐6 deficient mice resistance to EAM could be reversed by injecting IL‐6 around first immunization. IL‐6 expands dendritic cell and monocytic populations and ultimately leads to a robust T‐cell driven immune response in CFA immunized mice.

## Introduction

Adjuvants are known to enhance an immune response affecting innate and adaptive immunity 1, 2, 3, as well as the specificity of epitope recognition 4, 5. Experimental mouse models have contributed greatly to our understanding of the pathogenic mechanisms involved in human autoimmune diseases. Complete Freund's Adjuvant (CFA), emulsified with an antigen, is the most widely used method to induce an autoimmune disease in rodents 6. Examples of such diseases models include collagen‐induced arthritis (CIA), experimental autoimmune myocarditis (EAM), experimental autoimmune thyroiditis (EAT), experimental autoimmune encephalomyelitis (EAE), and experimental autoimmune uveitis (EAU), among others 7, 8, 9, 10.

In the animal models, adjuvant recapitulates the immune activation pathways elicited by infectious agents. Human myocarditis is often associated with previous enteroviral infection, and is modeled in susceptible strains of mice by coxsackie B3 infection 11, 12. In several animal models, the infectious agent can be replaced by an appropriate immunogenic stimulus, such as CFA, combined with the appropriate autoantigen. By using a myocarditigenic peptide emulsified in CFA, myocarditis can be reproducibly induced in the mouse without requiring viral infection 13, 14, 15, 16. In the present study, we use A/J and Balb/c mice that are both susceptible to EAM.

The induction of autoimmune diseases in rodents sometimes requires two subcutaneous injections of CFA. Repeated administration of CFA to animals can cause multiple injection‐site effects, the most often observed are on site granulomas and pain, which has necessitated justification for this method 17. For induction of EAM, two injections of CFA emulsified with murine myosin or myocarditigenic peptide are required 7. In this study, we demonstrate the differences between CFA and other adjuvants for inducing EAM.

To examine why CFA is essential for EAM development, we examined immune cells and their products, which have been shown to play a role in priming events during EAM induction. While CD4^+^ T cells are critical for the pathogenesis of EAM 18, other cell types are likely to exert priming functions in activating autoreactive T cells. Monocytes and macrophages comprise the bulk of inflammatory infiltrating cells in the heart during murine EAM and their phenotype is critically important in driving cardiac remodeling and fibrosis 19, 20, 21. DCs containing antigen are capable of initiating disease induction in several disease models. Transfer of DCs loaded with myocarditigenic peptide into naive mice has been shown to induce myocarditis 22.

Cytokines have also been shown to mediate EAM severity. To study the role of adjuvant, we focus on cytokines that are needed in priming events during EAM induction. The inflammatory cytokine IL‐6 is required for the development of EAM as IL‐6‐deficient knockout (IL‐6KO) mice are completely resistant to disease 23. IL‐6 has been extensively studied and shown to have pleiotropic activity on a broad range of immune cells. IL‐6 is produced by lymphoid and non‐lymphoid cells 24. It exerts effects on antigen presenting cells (APCs), B and T cells, as well as the induction of inflammatory cascades such as complement 24, 25. These effects include B cell activation and antibody responses, T cell activation and differentiation, differentiation of bone marrow precursors to macrophages and dendritic cells, as well as local effects on inflamed tissues 26, 27. Thus, the importance of IL‐6 on adjuvant efficacy is of special interest as IL‐6 may be a target to tune a desired immune response to immunization.

## Results

### Two doses of CFA are necessary for development of EAM

**Figure 1:**
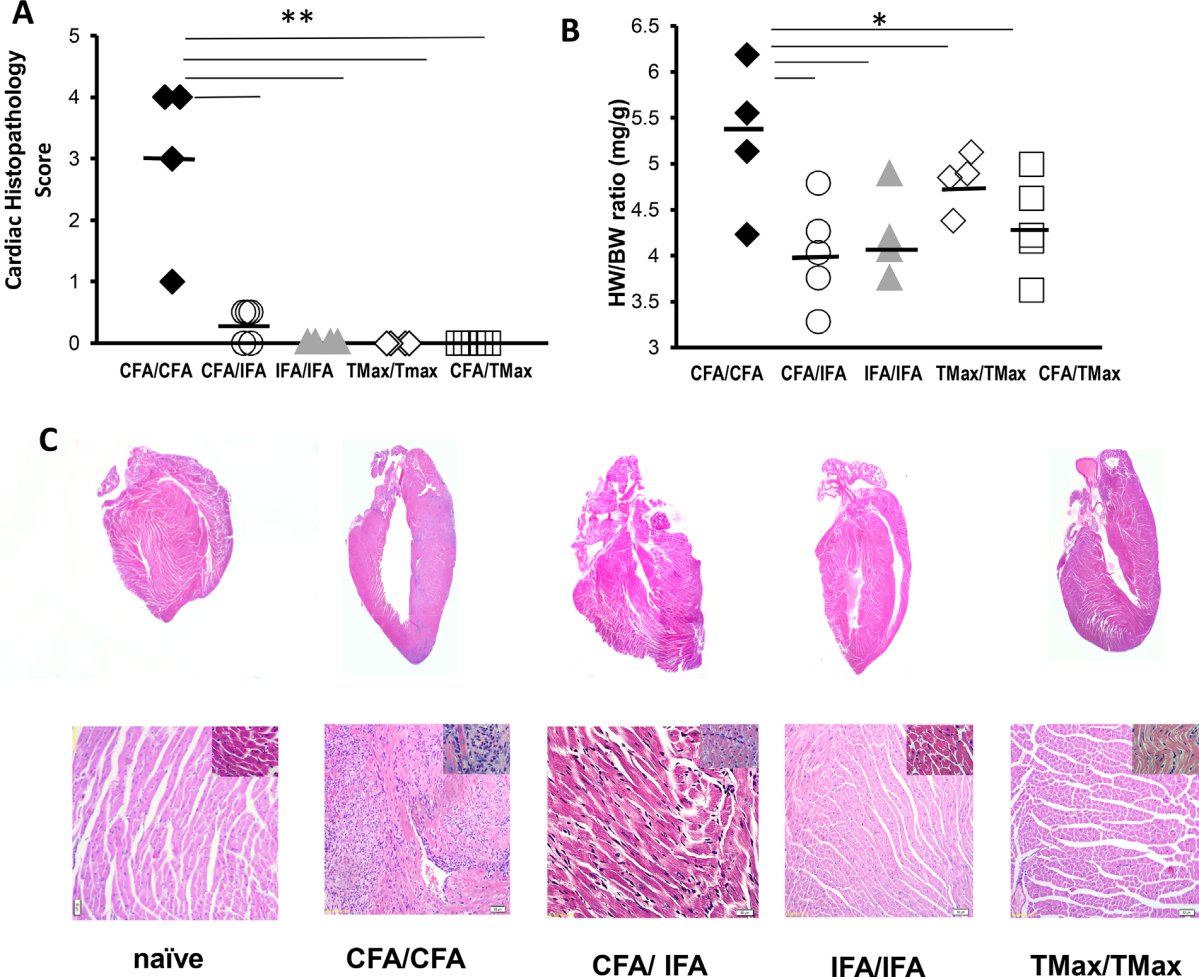
CFA is required for EAM induction. A/J mice were immunized on days 0 and 7 with myocarditogenic peptide emulsified in CFA or IFA or TiterMax. Mice were sacrificed 21 days post‐immunization. Data points represent individual mice. EAM was assessed by histopathology (A) as well as by heart weight/body weight ratio (B) Representative hematoxylin and eosin‐staining of cardiac sections from A/J mice immunized with CFA/CFA, CFA/IFA, IFA/IFA/, and TiterMax/TiterMax (C). All data are analyzed by one‐way ANOVA followed by Tukey's post‐test. * denotes *p* < 0.05, ** denotes *p* < 0.01 by Tukey post‐test for the groups indicated.

The protocol of EAM induction includes two immunizations with myocarditogenic peptide or myosin in CFA 7 days apart (days 0 and 7) 7, 28. We tested whether two supplemented CFA (5 µg/mL of *Mycobacterium tuberculosis*) doses on days 0 and 7 are required to induce myocarditis. Different combinations of CFA, IFA, and TiterMax emulsified with myocarditogenic peptide were injected subcutaneously on days 0 and 7. Only male mice were tested. Mice were sacrificed on day 21 and myocarditis severity was assessed by histology and relative heart weight. Only mice immunized with CFA on both days 0 and 7 developed substantial myocarditis as measured by histology (Fig. 1A and C) and relative heart weight (Fig. 1B). Other combinations of adjuvants were not sufficient to induce disease, including CFA followed by IFA (Fig. 1A–C). Thus, two doses of CFA are required for the induction of EAM.

### CFA enhances antigen‐specific antibody response

**Figure 2:**
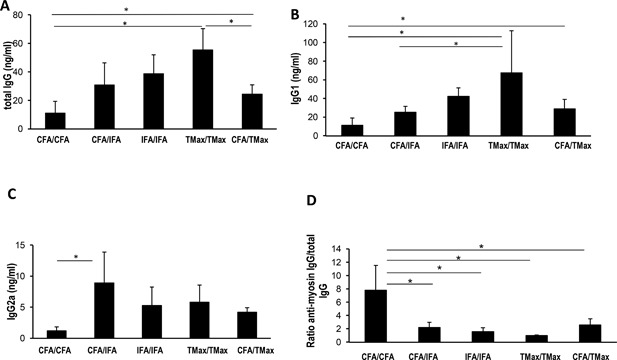
Total IgG (A), IgG1 (B), IgG2a (C), and ratio of total IgG to MyHCα_339–352_ IgG (D) were measured in all groups (CFA/CFA, CFA/IFA, IFA/IFA/, and TiterMax/TiterMax). All values are in ng/ml. Bars represent mean. *N* = 4–5 mice/group. All data analyzed by one‐way ANOVA followed by Tukey's post‐test. * denotes *p* < 0.05 by Tukey post‐test for the groups indicated.

Knowing that CFA was required to induce EAM, we examined other immunological properties of the CFA/CFA group. To assess the ability of each adjuvant or combination of adjuvants to induce antibody responses, we measured levels of total as well as MyHCα_339–352_ specific IgG, IgG1, IgG2a, and IgG2b levels on day 21 (Fig. 2A–D and not shown). IFA or TMax immunized animals had significantly increased levels of total IgG as well as IgG1 and IgG2a compared to CFA immunized mice (Fig. 2A–C). However, the ability of adjuvants to induce a non‐antigen specific antibody response was inversely related to their induction of antigen‐specific immune responses. An anti‐MyHCα_339–352_ response was seen when at least one injection of CFA was used, compared to mice immunized with IFA or TMax without any CFA. Figure 2D shows that the ratios of MyHCα_339–352_ specific IgG to total IgG were significantly increased in the CFA/CFA group compared to all other groups. Therefore, while TMax and IFA induced strong non‐antigen specific antibody responses, two CFA injections were required to induce significant autoantigen‐specific antibody responses in EAM. Although EAM is not an antibody‐driven disease 29, the presence of antigen‐specific responses in CFA versus IFA or TMax indicate that CFA drives a specific, autoantigen‐focused B cell response, whereas IFA and TMax elicit a broad population of antibodies many of which are not specific for the autoantigen.

### CFA expands the myeloid population in the spleen at day 21

**Figure 3:**
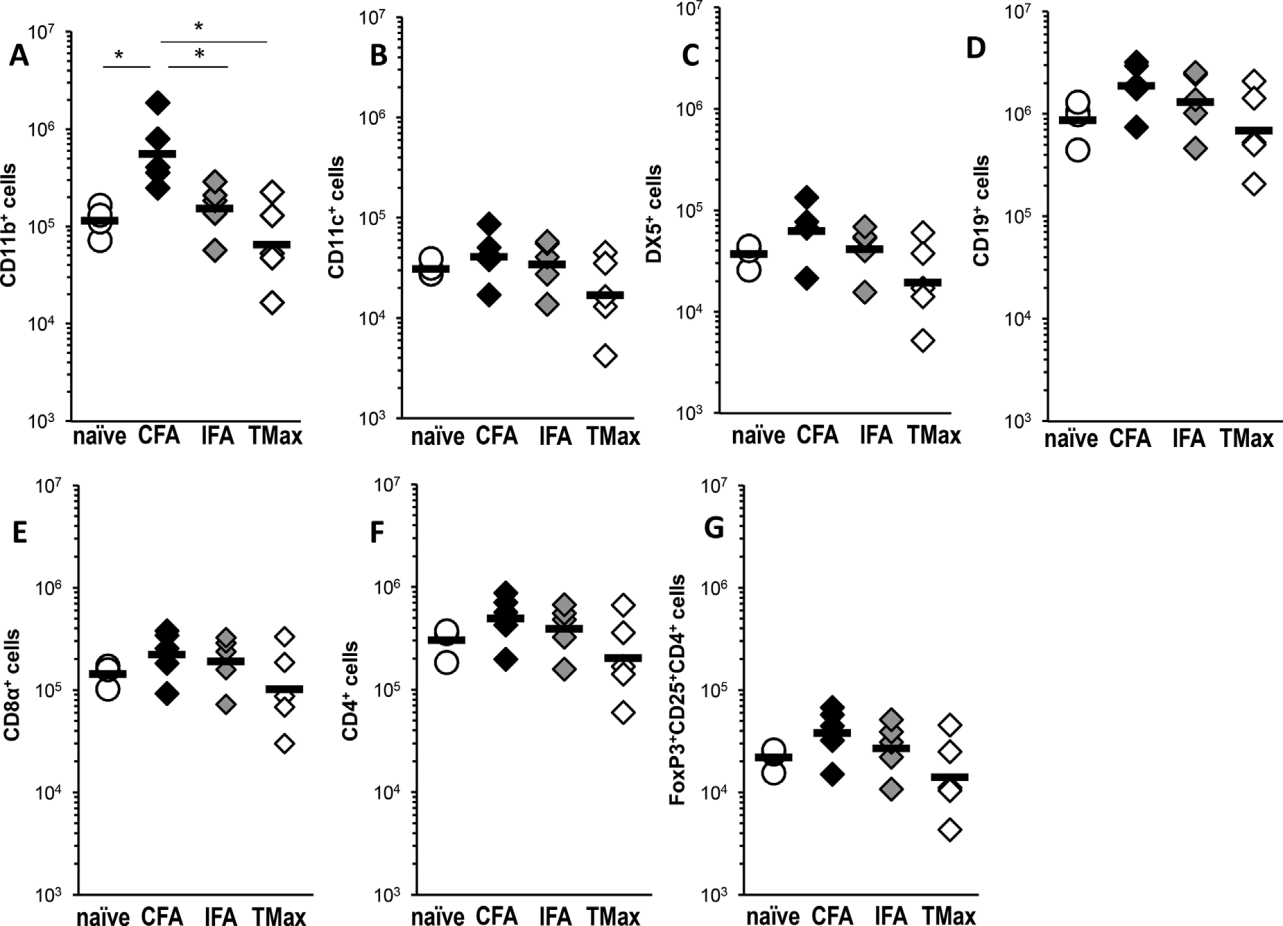
CFA expands the myeloid population in the spleen at day 21. A/J mice were immunized on day 0 and 7 with myocarditogenic peptide emulsified in CFA or IFA or TiterMax. Total number of CD11b^+^ monocytes (A), CD11c^+^ dendritic cells (B), CD4^+^ T cells (C), CD19^+^ B cells (D), DX5^+^ NK cells (E), CD8^+^ T cells (F), or CD4^+^CD25^+^FoxP3^+^ Tregs (G) in the spleens on day 21 of EAM is shown. Spleens from naive animals were used as controls. Data points represent individual mice. Bars represent mean. *N* = 4–5 mice/group. All data are analyzed by one‐way ANOVA followed by Tukey's post‐test. * denotes *p* < 0.05 by Tukey post‐test for the groups indicated.

We further sought to identify the underlying mechanisms requiring CFA as opposed to other adjuvants in EAM. We analyzed differences in the composition of immune cells in the spleen 21 days after immunization with CFA, IFA or TMax. We first investigated gross immune population expansion in the spleen in order to guide our investigation into the heart tissue in subsequent experiments. CFA induced a significant increase in the number of CD11b^+^ monocytes compared to IFA and TMax groups (Fig. 3A). No differences were observed in number of CD11c^+^ dendritic cells, CD4^+^ T cells, CD19^+^ B cells, DX5^+^ NK cells, CD8^+^ T cells, or CD4^+^CD25^+^FoxP3^+^ Tregs (Fig. 3B–G). Thus, CFA dramatically expanded monocytic populations in the spleen without affecting proportions of other cell types on day 21 of EAM.

### CFA expands the myeloid population in the heart at day 21

**Figure 4:**
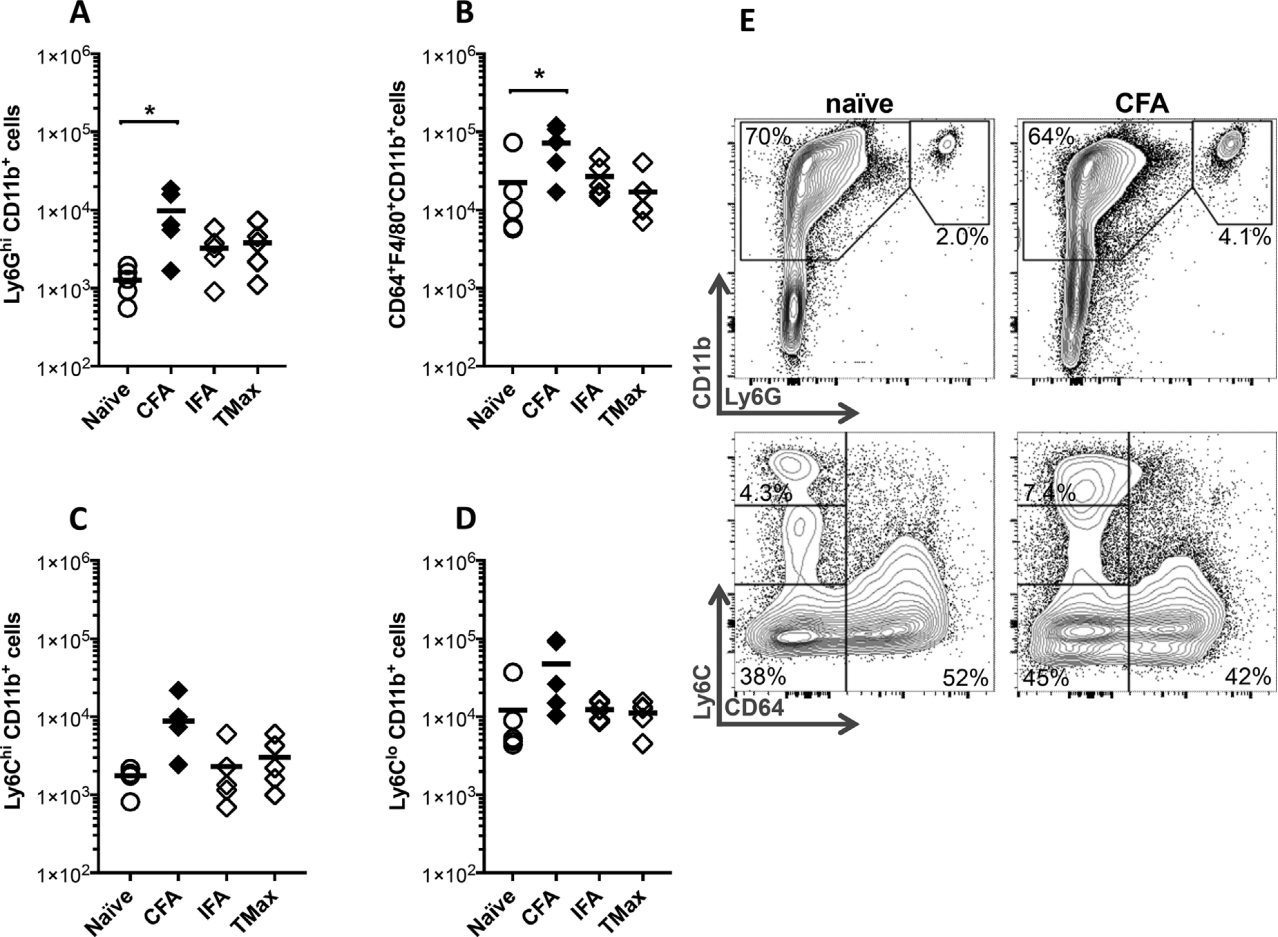
CFA expands the myeloid population in the heart at day 21. BALB/c mice were immunized on days 0 and 7 with myocarditogenic peptide emulsified in CFA or IFA or TiterMax. Cell population were analyzed from their hearts on day 21 of EAM. Total number of neutrophils Ly6G^hi^CD11b^+^ (A), macrophages CD64^+^F4/80^+^CD11b^+^ (B), inflammatory monocytes Ly6C^hi^CD11b^+^ (C), and non‐inflammatory monocytes Ly6C^lo^CD11b^+^ (D) is shown. Representative flow cytometry gating of these myeloid populations is shown (E). Data points represent individual mice. Bars represent mean. *N* = 4–5 mice/group. All data are analyzed by one‐way ANOVA followed by Tukey's post‐test. * denotes *p* < 0.05 by Tukey post‐test for the groups indicated.

Next, we analyzed differences in the composition of different types of myeloid cells in the heart 21 days after immunization with CFA, IFA, or TMax (Fig. 4A–E). Example of our gating strategy of neutrophils and monocytes and macrophages is in Figure 4E. Immunization with CFA induced a significant increase in the numbers of neutrophils Ly6G^hi^CD11b^+^ (Fig. 4A), macrophages CD64^+^F4/80^+^CD11b^+^ (Fig. 4B), inflammatory monocytes Ly6C^hi^CD11b^+^ (Fig. 4C), and non‐inflammatory monocytes Ly6C^lo^CD11b^+^ (Fig. 4D). Thus, by day 21, CFA dramatically expanded all myeloid populations in the heart.

### CFA stimulates monocytes and dendritic cells expansion and IL‐1β and IL6 production in the spleen at day 3

**Figure 5:**
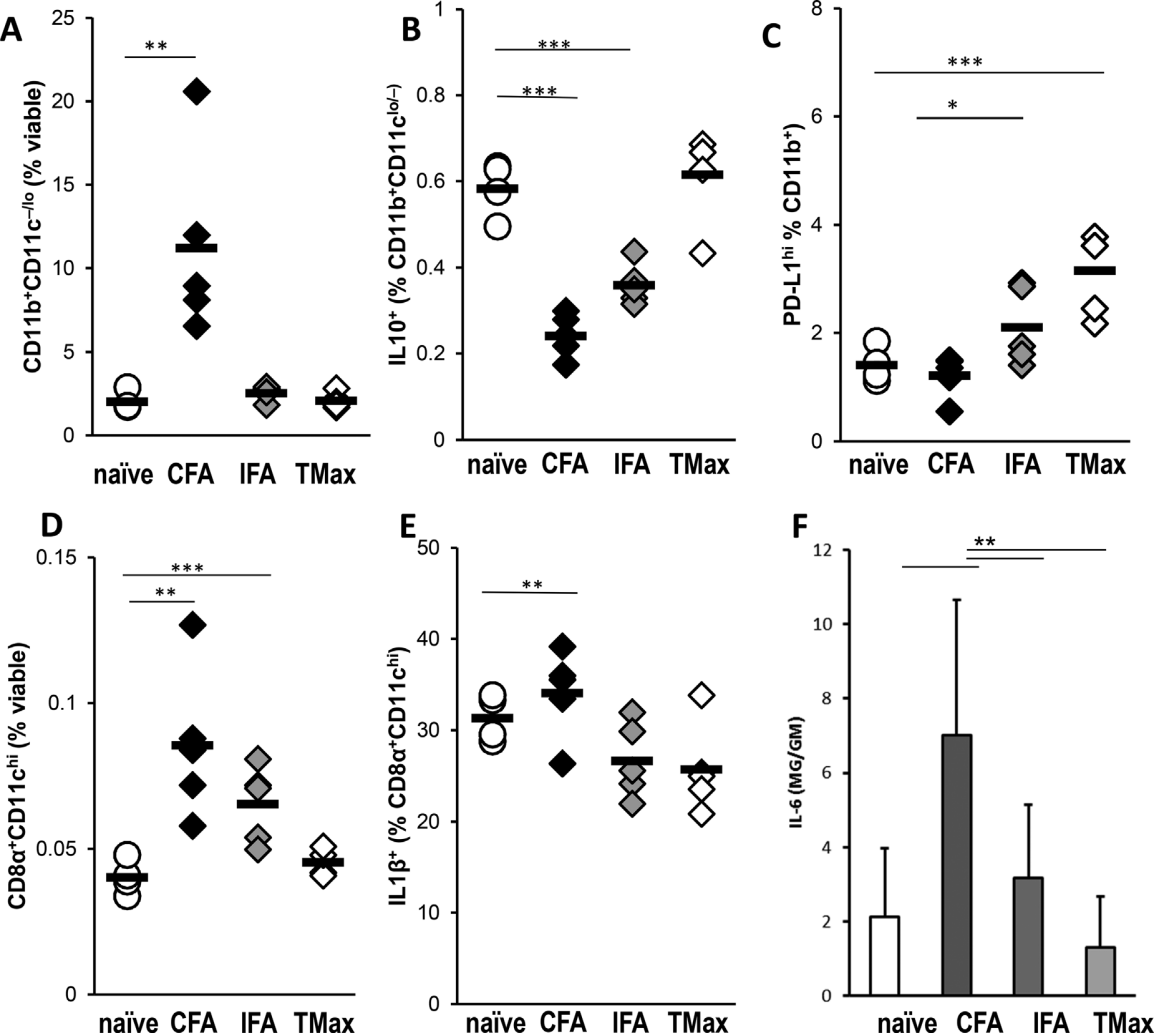
CFA stimulates an inflammatory environment in the spleen at day 3. A/J mice were immunized on day 0 with myocarditogenic peptide emulsified in CFA or IFA or TiterMax. Total number of CD11b^+^CD11c^−/lo^ monocytes in the spleen 3 days after the first injection with the shown adjuvant (A). CD11b^+^CD11c^−/lo^ monocytes production of IL‐10 (B), expression of PD‐L1 (C). Total numbers of conventional CD8α^+^CD11c^+^ dendritic cells (D) IL‐1β production by CD8α^+^CD11c^+^ dendritic cells (E). IL‐6 production was analyzed from spleens of Balb/c mice after on day 3 of immunization with the appropriate adjuvant (F). Spleens from naive animals were used as controls. Data points represent individual mice. Bars represent mean. *N* = 4–5 mice/group. All data are analyzed by one‐way ANOVA followed by Tukey's post‐test. * denotes *p* < 0.05, ** denotes *p* < 0.01, *** denotes *p* < 0.001 by Tukey post‐test for the groups indicated.

Next, we wanted to examine whether expansion of monocytes in the periphery was already apparent during the priming period following CFA immunization. We immunized mice with one dose of CFA, IFA, or TMax and analyzed splenocyte populations on day 3. CFA immunized mice accumulated greater numbers of CD11b^+^CD11c^−/lo^ monocytes in the spleen 3 days after the first injection, compared to IFA and TMax immunized animals (Fig. 5A). Those monocytes produced lower levels of the anti‐inflammatory cytokine IL‐10 (Fig. 5B), as well as diminished expression of the negative costimulator PD‐L1 (Fig. 5C). Additionally, CFA immunized mice had higher total numbers of conventional CD8α^+^CD11c^+^ dendritic cells (Fig. 5D) that were producing IL‐1β (Fig. 5E) on day 3. Another important cytokine produced by monocytes and other innate cells is IL‐6. We assessed the levels of IL‐6 in the spleen of mice immunized with CFA, IFA, or TMax on day 3, and naïve controls by ELISA. CFA immunized animals had significantly increased levels of IL‐6 in their spleens on day 3 of EAM compared to controls (Fig. 5F). Together, these results indicate that CFA induces expansion of monocytes and dendritic cells in spleen 3 days after immunization. Moreover, CFA induced high levels of IL‐1β and IL6 production.

### Recombinant IL‐6 treatment partially restores susceptibility to EAM in IFA immunized mice

**Figure 6:**
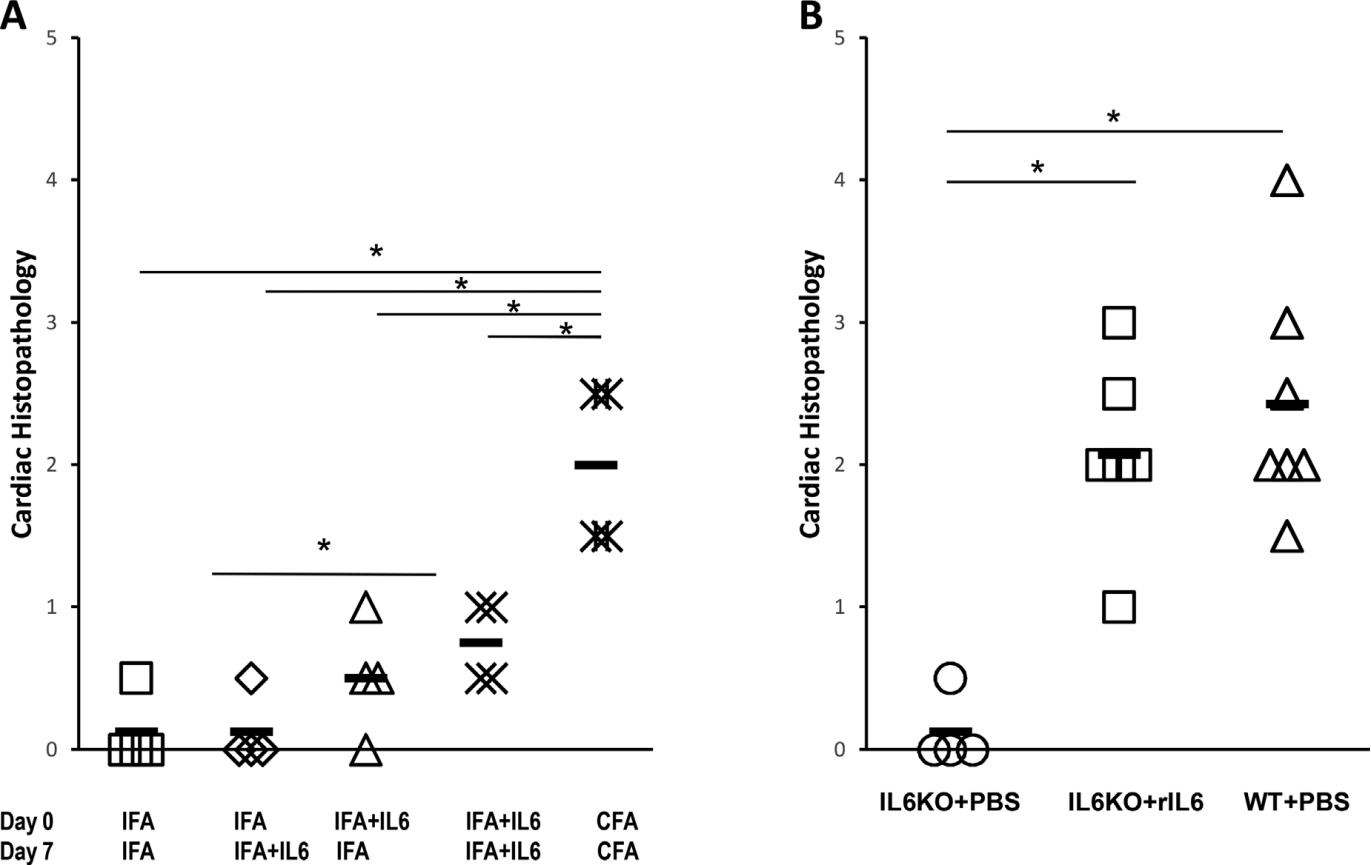
IL‐6 is required for the initial response to immunization in order to induce EAM. EAM was induced in WT A/J mice immunized with IFA or CFA, alone or with recombinant IL6 treatment on the day indicated (*x*‐axis). Mice were sacrificed 21 days post‐immunization. Myocarditis severity was assessed by histopathology (A). *N* = 4 mice/group. EAM was induced in WT Balb/c mice or IL‐6KO mice on Balb/c background. On days −1, 0, 1, 2 mice were treated with either PBS or 50 ng recombinant IL‐6 *iv*. Mice were sacrificed 21 days post‐immunization (B). Myocarditis severity was assessed by histopathology. *N* = 6 mice/group. Data points represent individual mice. Bars represent mean. Data are analyzed by one‐way ANOVA followed by Tukey's post‐test. * denotes *p* < 0.05 by Tukey post‐test for the groups indicated.

We have noted above that the early expansion of monocyte population was accompanied by higher levels of IL‐6 3 days after immunization with CFA (Fig. 5F). In order to ascertain whether pathogenic responses to immunization with CFA were mediated by IL‐6, we treated immunized A/J mice with recombinant intravenous IL‐6 to examine whether IL‐6 was sufficient to replace mycobacterial‐derived innate immune signals. Histopathologic examination of mice hearts demonstrated that mice immunized with IFA and treated with recombinant IL‐6 at the day 0 developed myocarditis (Fig. 6A). Mice immunized with IFA alone failed to develop cardiac inflammation, whereas mice immunized with CFA developed severe myocarditis (Fig. 6A). This finding demonstrates that recombinant IL‐6 treatment partially restores susceptibility to EAM in otherwise EAM‐resistant mice. Therefore, IL‐6 is partially responsible cardiopathogenic susceptibility to adjuvant during autoreactive priming.

### IL‐6 is only required for the initial response to immunization in order to induce EAM

We have confirmed that IL‐6 is important for the response to adjuvant in inducing subsequent pathogenic responses against heart antigen. We then sought to identify the critical window in which IL‐6 is absolutely required for EAM disease induction. It has been reported that IL‐6KO mice are resistant to EAM 23. To determine the role IL‐6 plays in the development of EAM in response to immunization, IL‐6KO mice were treated with PBS or recombinant IL‐6 *iv* around the time of their first immunization, on days −1, 0, 1, and 2, in order to restore their susceptibility to EAM. Balb/c mice were used in all experiments involving IL‐6 since IL‐6 KO mice are not available on A/J background. We have first established that response to different adjuvants in Balb/c mice is similar to A/J mice in Supplemental Figure S1 and that CFA is essential for EAM induction also for Balb/c mice. Histopathology examination of mouse hearts showed that IL‐6KO mice receiving recombinant IL‐6 *iv* developed myocarditis comparable to WT mice, whereas IL6KO mice were resistant to EAM (Fig. 6B). This result demonstrates that IL‐6 is required only at the initial priming phase of disease, and is subsequently dispensable for EAM development as the initial recombinant IL6 treatment was sufficient to restore disease susceptibility.

### IL‐6 leads to differential dendritic cell profiles in the draining lymph node following immunization

**Figure 7:**
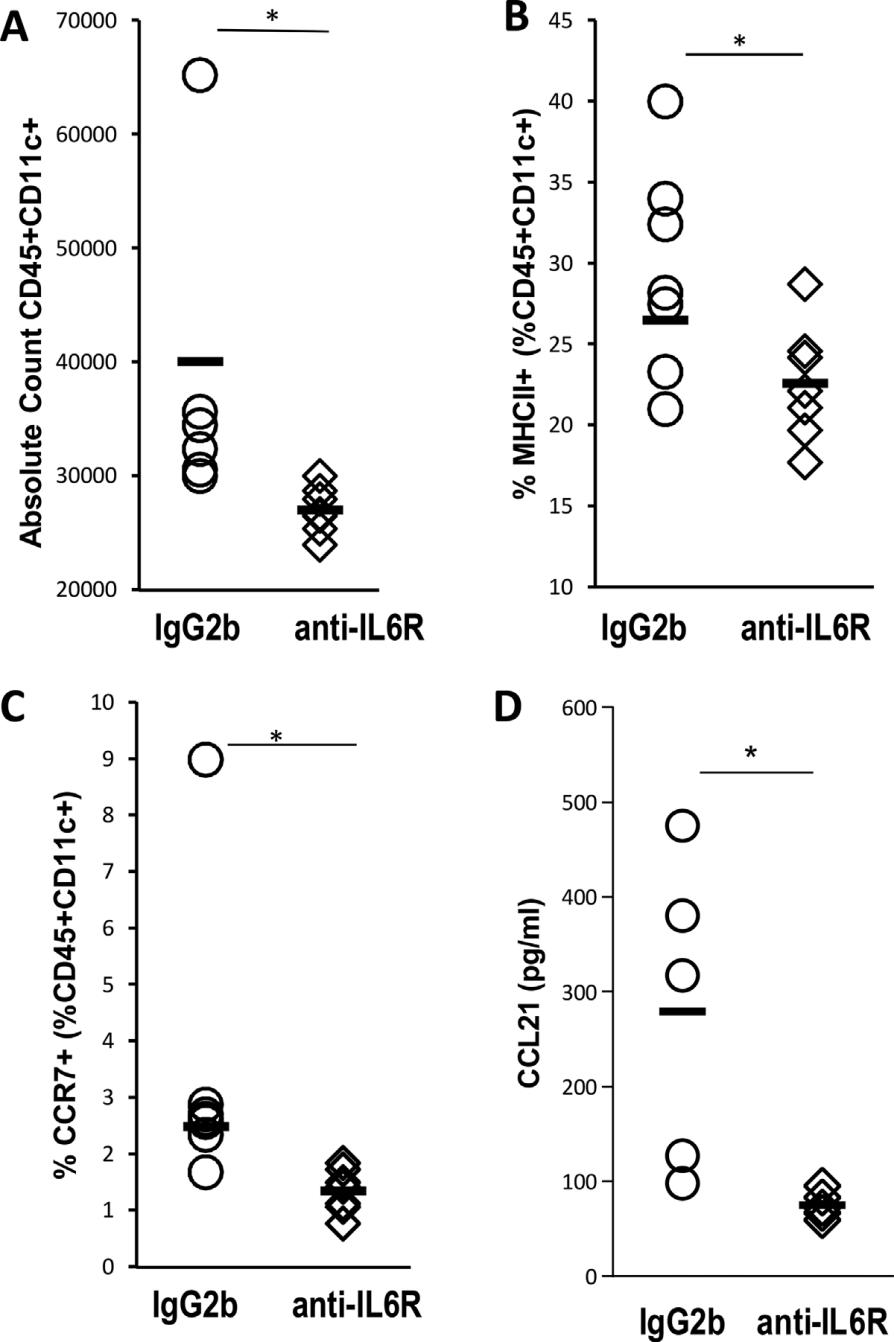
IL6 is required in order to induce inflammatory DC trafficking to the draining LN following immunization. EAM was induced in WT Balb/c mice treated with isotype control IgG2b antibodies or anti‐IL‐6‐receptor antibodies on days −3 and 0. Mice were sacrificed 3 days post‐immunization. Response to immunization was assessed in the draining lymph node of the immunization site by flow cytometry. Total number of CD11c^+^ dendritic cells in the inguinal lymph nodes draining sites of immunization (A). Expression of MHC Class II by CD11c^+^ dendritic cells (B). Expression of CCR7 by CD11c^+^ dendritic cells (C). Data were analyzed by Student's *t*‐test. Data are representative of three independent experiments. Total lymph node homogenates were assessed for CCL21 levels by ELISA (D). Data are analyzed by Student's *t*‐test. *N* = 7 mice/group. Data points represent individual mice. Bars represent mean. * denotes *p* < 0.05

Knowing that IL‐6 is required during a limited timeframe for disease induction led us to investigate the mechanism by which IL‐6 alters the immune response to adjuvant. In order to more precisely define the role that IL‐6 plays in response to immunization, we studied the immune response in the lymph nodes draining the immunization site 3 (inguinal lymph nodes) days following a single immunization. BALB/c mice were pre‐treated with either control or anti‐IL6R antibodies (IL6‐blocking antibody, MR‐16, Chugai) on days −3 and 0 then immunized in the hind limb on day 0, and sacrificed 3 days post‐immunization. Flow cytometric analysis showed that mice treated with anti‐IL‐6R antibodies had significantly lower number of CD11c^+^ dendritic cells in the inguinal lymph nodes draining sites of immunization (Fig. 7A). These dendritic cells had lower expression of MHC Class II as compared to untreated mice (Fig. 7B). There were no differences in any other population examined, including total CD45^+^ cells, CD11b^+^, CD19^+^, or CD4^+^ cells (data not shown). These data demonstrate that IL‐6 is important for the accumulation and maturation of dendritic cells in the draining lymph nodes, in association with subsequent induction of EAM.

### IL‐6 induces dendritic cell migration to the draining lymph node through DC CCR7^+^ expression and LN CCL21 expression

Because we found IL‐6 induced accumulation of dendritic cells in the lymph nodes draining the immunization site, we further investigated whether IL‐6 is able to induce trafficking of those cells to lymph nodes. We examined the expression of chemokine ligands in lymph node homogenates, as well as expression of chemokine receptors on dendritic cells. Interestingly, the levels of CCR7 on lymph node dendritic cells were decreased in mice treated with anti‐IL6R (Fig. 7C). CCL21 has been shown to direct migration through CCR7; this pathway has been further demonstrated to be important in rheumatoid arthritis 30. Additionally, it has been demonstrated that CCL21 is critical in regulating dendritic cell homeostasis and function through CCR7, in a manner upregulated by inflammatory cytokines 31, 32. Therefore, we further investigated the expression of CCL21 in the draining lymph node. In agreement with these other studies, we found by ELISA that the production of CCL21 was also decreased in lymph nodes tissue homogenate of the mice treated with anti‐IL6R antibodies (Fig. 7D). The production of other cytokines and chemokines in the draining lymph node showed few other statistically significant changes (data not shown). Together, these experiments illustrate that IL‐6 promotes dendritic cells trafficking to the draining lymph node through the up regulation of CCR7 on dendritic cells, as well as increased expression of CCL21 in the draining lymph node. This is of particular importance because it suggests that IL‐6 production at the site of immunization alters the local cytokine and chemokine environment allowing dendritic cell trafficking to the lymph node.

## Discussion

CFA is required in most immunization‐induced animal models such as collagen‐induced arthritis (CIA), experimental autoimmune encephalomyelitis (EAE), experimental autoimmune thyroiditis (EAT), experimental autoimmune uveitis (EAU), and EAM 7, 9, 10, 15, 28. In these models, the adjuvant provides the required immune activation to initiate disease development when accompanied by an appropriate autoantigen. It was originally shown that thyroiditis could be induced in mice by administering CFA plus thyroglobulin, whereas IFA was not a sufficient adjuvant for thyroid lesions 33, 34. These initial studies not only demonstrated how autoimmunity could be modeled in animals, but how different immune triggers led to vastly different disease outcomes. The continued use of CFA as the inducing adjuvant is due to the fact that no other adjuvant has been able to provide consistent immunopathogenicity 17.

CFA emulsified with myosin or myocarditogenic peptide in two injections 7 days apart is the classical protocol to induce EAM 35, 36, 37, 38, 39, 40, 41, 42. In this study, we have determined that two CFA injections are required to Induce EAM in both A/J and BALB/c male mice and showed some of the mechanisms of how CFA contributes to disease induction. We have established that CFA cannot be replaced with IFA or TiterMax for either immunization. In concordance with these findings, in the both the EAU and EAE models, stimulation of multiple TLR pathways was necessary to induce autoimmunity; individual TLR ligands were unable to substitute for CFA 43, 44. Additionally, it has been shown that TiterMax is ineffective at T cells responses 45.

Although B cells are not required for EAM induction 29, antibodies may contribute to disease severity and levels of anti‐myosin antibodies correlate well with disease severity 46, 47. We have observed increased percentages of CD19^+^ B cells after mice were immunized with only TiterMax or IFA, compared to mice immunized at least one time with CFA. Additionally, total IgG and IgG subclasses were increased in serum across all subclasses when mice were immunized with TiterMax or IFA. Adjuvants like alum have been shown to induce antibody responses comparable to CFA 48. When we compared the levels of myocarditogenic‐peptide specific antibodies, we found that CFA immunized mice produced mainly anti‐myocarditogenic peptide specific antibodies as shown by anti‐myosin IgG/total IgG ratio. Thus, IFA and TiterMax increase the mobilization of B cells, and induce polyclonal antibody production; however, many of these antibodies were not specific to myocarditogenic peptide. In contrast, CFA was the most effective at eliciting antigen‐specific antibodies.

EAM is driven by T cells; however, monocytes, macrophages, and granulocytes are the most abundant cell populations to infiltrate the heart 19. This is important because CFA did not increase percentages of CD4^+^ or CD8^+^ T cells, or CD4^+^ CD4^+^CD25^+^FoxP3^+^ Tregs; however, it did increase the relative proportion of CD11b^+^F4/80^−^monocytes and CD11b^+^Gr1^+^ granulocytes. The spleen myeloid cells expansion was seen early after the immunization with CFA and the myeloid cells expressed lower levels of the regulatory cytokine IL‐10 and the inhibitory molecule PD‐L1. Additionally, neutrophils, Ly6C^hi^ monocytes, Ly6C^low^ monocytes and CD64^+^F4/80^+^ macrophages were all expanded in the heart after CFA immunization compared to IFA or TiterMax immunized mice. This lends support to the notion that one of the mechanisms by which CFA contributes to EAM induction is through a proinflammatory environment, mobilizing monocytes, and granulocytes.

Dendritic cells are also important for disease induction as cardiac antigen‐loaded DCs induce autoimmune myocarditis when they were activated and transferred into naïve mice 22. Previous studies have shown that dendritic cells and key cytokines regulate the fate of T cells in myocarditis development in the Lewis rat 49. In this study, we demonstrate that CFA expanded monocytes, and IL‐1β‐producing CD8a^+^CD11c^+^ DCs in the spleen following immunization.

Many studies have shown that EAM is a T‐cell‐mediated disease 50, 51, 52, 53, 54. The necessity of CFA as an adjuvant to induce disease suggests that strong activation of the innate immune system is essential for EAM development 55, 56, 57. CFA, which contains numerous microbial TLR ligands (such as TLRs 2, 4, and 9), is a potent inducer of a Th1‐type immune response. Although it contains TLR ligands, CFA can elicit a robust antibody response to T‐cell‐dependent antigens in TLR‐deficient mice 58, 59. This T‐cell‐dependent, antigen‐specific antibody response elicited by CFA is not TLR dependent because MyD88^−/−^ mice developed antibody responses comparable to WT mice 60. Therefore, induction of EAM by CFA utilizes additional innate activation triggers.

Many inflammatory cytokines promote myocarditis development in animals 61, 62, 63, 64, 65. Mice that lack IL6 signaling are protected from EAM 23, 66. Importantly, CFA responses are dependent on IL6 for full adjuvant capacity 67. We show here that CFA increases IL6 levels in the spleen following immunization. Additionally, we establish that the increased immunogenicity of CFA compared with IFA is partially due to the induction of IL‐6; the efficacy of IFA immunization can be partially restored by the administration of recombinant IL‐6. We have demonstrated the requirement of IL‐6 in EAM induction is limited to the first immunization as recombinant IL‐6 administration to EAM‐resistant IL‐6 KO mice surrounding the first immunization completely restores susceptibility in those mice. Finally, we have shown that IL‐6 induces CCR7^+^ dendritic cell trafficking to the local lymph node, in conjunction with increased CCL21 expression by the local lymph node. CCL21 expression is upregulated in rheumatoid arthritis wherein CCL21 is the critical CCR7 ligand mediating migration, whereas CCL19 is redundant. 30, 31. It has also been shown that lymphatic CCL21 expression can be upregulated by inflammatory cytokines 32, confirming our observation that IL‐6 mediates expression of both CCL21 ligand and its CCR7 receptor. Together, these studies corroborate that EAM induction is driven by dendritic cells responding to inflammatory cytokines, in addition to heart reactive T cells 22, 63.

In conclusion, we have shown that CFA used with myocarditogenic peptide twice is required to induce EAM in both A/J and Balb/c mice. Although IFA and TiterMax induce antibody responses, only CFA preferentially induced autoantigen‐specific responses. CFA expands monocytes in the heart and in the spleen and those monocytes in the spleen make less IL‐10 as well as more IL‐6. We have shown that IL‐6 signaling is required during short window around primary immunization to induce EAM and that IL‐6 deficient animals resistance to EAM could be reversed by injecting IL‐6 around first immunization. In addition, the immune response to IFA can be augmented by recombinant IL‐6 administration. Finally, we have shown that IL‐6 enables CCL21‐mediated trafficking of CCR7^+^ dendritic cells to the local lymph node, expands dendritic cell and monocytic populations and ultimately leads to a robust T‐cell driven immune response not found in adjuvants such as IFA or TiterMax.

## Materials and Methods

### Mice

A/J, BALB/c, and IL6KO BALB/c male mice were obtained from the Jackson Laboratory (Bar Harbor, ME) and maintained in the Johns Hopkins University School of Medicine SPF animal facility. The facility maintains standard light/dark cycles and feeds ad libitum. All experiments were conducted on 6–8‐week‐old male mice and in compliance with the Animal Welfare Act and the principles set forth in the Guide for the Care and Use of Laboratory Animals. All methods and protocols involving mice were approved by the Animal Care and Use Committee of the Johns Hopkins University. Invasive procedures were performed under Avertin (Sigma–Aldrich, St. Louis, MO) anesthesia. No animals died prior to experiment endpoint. Animals were euthanized with Avertin and cervical dislocation, according to protocols approved by the Animal care and Use Committee of the Johns Hopkins University.

### Induction of EAM and use of different adjuvants

For the induction of EAM, we used the myocarditogenic peptide derived from the sequence of the murine cardiac myosin heavy chain (DSAF DVLS FTAE EKAG VYK for A/J mice and Ac‐SLKLMATLFSTYASAD for BALB/c mice) 68, 69 commercially synthesized by fMOC chemistry and purified by HPLC (Genscript, Picataway, NJ). On days 0 and 7, mice received subcutaneous injections peptide emulsified in CFA (Sigma, St. Louis, MO) supplemented with 5 mg/mL of *Mycobacterium tuberculosis,* strain H37Ra (Difco, Detroit, MI). Alternatively, we replaced CFA with the same amount of IFA (Sigma) or TiterMax Gold Adjuvant (Sigma) emulsified these adjuvants with myocarditogenic peptide in same manner as CFA. On day 0, all mice additionally received 500 ng of pertussis toxin *ip* (List Biologicals, Campbell, CA). Individual experiments were conducted at least three times with five mice per group.

### Histopathology

Mice were evaluated for the development of EAM at the peak of disease on day 21. Heart tissues were fixed in 10% phosphate‐buffered formalin. A total of 5 μm sections were cut longitudinally and stained with hematoxylin and eosin. Myocarditis severity was evaluated by histopathologic microscopic approximation of the percent area of myocardium infiltrated with mononuclear cells or fibrosis determined from five sections per heart according to the following scoring system: grade 0 − no inflammation; grade 1 − less than 10% of the heart section is involved; grade 2 − 10–30%; grade 3 − 30–50%; grade 4 − 50–90%; grade 5 − more than 90%. Two independent researchers scored slides separately in a blinded manner.

### Antibodies to cardiac myosin

Mice were bled on days 0 and 21 from the retro‐orbital venous plexus using heparinized capillary tubes. Serum levels of myosin‐reactive antibodies IgG and its subclasses were determined using microtiter plates coated with 0.5 µg MyHCα_614‐629_ or cardiac myosin incubated with phosphatase‐conjugated isotype‐specific secondary antibodies.

### Cytokine and chemokine ELISA

Lymph node, heart, or spleen was snap frozen on dry ice immediately after resection and stored at −80°C until homogenized in MEM + 2% FBS, debris cleared by centrifugation, and stored at −80°C until used in ELISA. Cytokine levels were measured using calorimetric ELISA kits (R&D Systems and Sigma) in homogenized heart supernatants, spleen supernatants, or lymph nodes supernatants. Heart cytokine were expressed as pg/g of heart tissue.

### Flow cytometry

Splenocytes or lymph node cells were extracted into single cell suspension in 1× PBS, and RBCs lysed by incubation in ACK lysis buffer (Biofluids), single cell suspensions were passed through 100 µm cell strainer (BD Falcon). Cells were washed and FcγRII/III blocked with αCD16/32 (eBiosciences). Surface markers were stained with fluorochrome‐conjugated mAbs toCD3, CD4, CD69, CCR7, IL7Rα, CD62L, CD44, CD45, CD103, CD25, CD8α, CD11b, CD11c, CD19, CD62L, CD80, CD86, CD117 (c‐kit), DX5, F4/80, MHC Class II (I‐A/I‐E), (eBiosciences, BD Pharmingen, Biolegend, AbD Serotec). Treg cells were further stained by intracellular staining of Foxp3 with a kit according to manufacturer's instructions (eBioisciences). Samples were acquired on the LSR II quad‐laser cytometer running FACSDiva (BD Immunocytometry).

### Statistical analyses

Statistical analysis was performed by one‐way ANOVA analysis. Normally distributed data on continuous parametric axes were analyzed with the 2‐tailed Student's *t*‐test. Values of *p *< 0.05 were considered statistically significant.

## Conflicts of Interest

The authors have no financial conflicts of interest to disclose.

## Supporting information

Additional supporting information may be found in the online version of this article at the publisher's web‐site

**Figure S1**. Histopathological examination of EAM in the hearts of immunized Balb/c mice. Representative hematoxylin and eosin‐staining of cardiac sections from A/J mice immunized with CFA/CFA, IFA/IFA/ and TiterMax/TiterMax. Severity of EAM and cardiac inflammation is highest in the CFA/CFA immunized group similar to what was observed in the A/J mice.
