# Supplementary figures and images for: Complete Freund's adjuvant induces experimental autoimmune myocarditis by enhancing IL‐6 production during initiation of the immune response
Source: Immun Inflamm Dis. 2017 Mar 13;5(2):163–76. doi: 10.1002/iid3.155 (PMC5418134; doi:10.1002/iid3.155)

## Slide 1
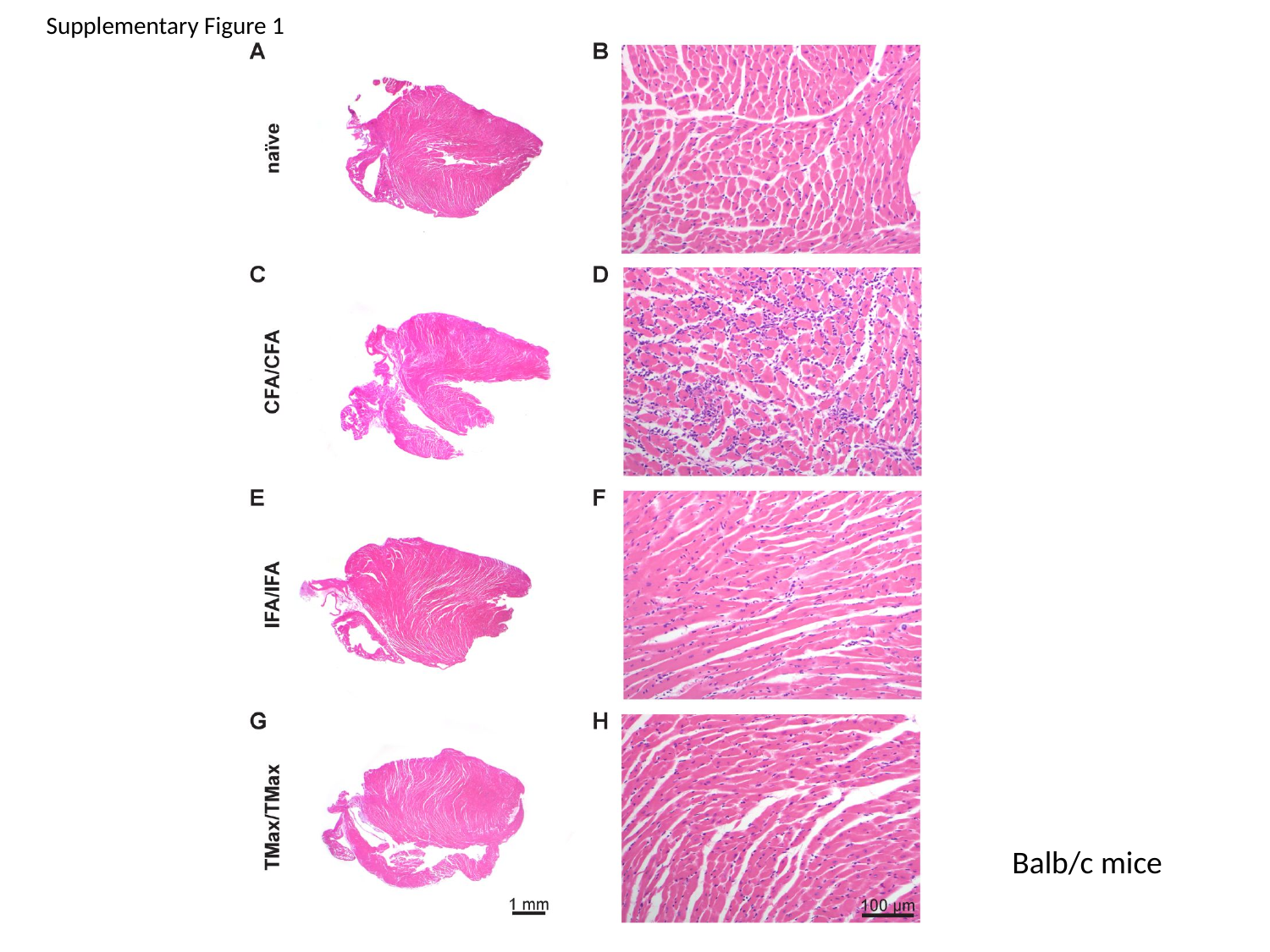

Supplementary Figure 1
Balb/c mice

Supplement: Supplementary file 1 — Figure S1. Histopathological examination of EAM in the hearts of immunized Balb/c mice. Representative hematoxylin and eosin‐staining of cardiac sections from A/J mice immunized with CFA/CFA, IFA/IFA/ and TiterMax/TiterMax. Severity of EAM and cardiac inflammation is highest in the CFA/CFA immunized group similar to what was observed in the A/J mice. [file IID3-5-163-s001.pptx]
